# METTL3-mediated m6A modification of KIF3C-mRNA promotes prostate cancer progression and is negatively regulated by miR-320d

**DOI:** 10.18632/aging.203541

**Published:** 2021-09-19

**Authors:** Honggui Ma, Facai Zhang, Quliang Zhong, Jianquan Hou

**Affiliations:** 1 The Department of Urology, The First Affiliated Hospital of Soochow University, Suzhou 215031, Jiangsu, China; 2 The Department of Urology, The Affiliated Hospital of Guizhou Medical University, Guiyang 550009, Guizhou, China

**Keywords:** METTL3, m6A, KIF3C, miR-320d, prostate cancer

## Abstract

The occurrence of distant metastasis is one of the leading causes of death in patients with prostate cancer (PCa). It is confirmed that kinesin protein is associated with a variety of malignancies, and the KIF3 family is related to cancer, but the relationship between KIF3C and prostate cancer is not clear. Our experiments have confirmed that KIF3C is highly expressed in prostate cancer tissues and cell lines. Further, functional tests have proven that KIF3C can promote the growth migration and invasion of PCa. We used Starbase 3.0 to discover that methyltransferase like 3 (METTL3) interacts with KIF3C. Our hypothesis and experiments concluded that METTL3 induced m6A modification on KIF3C, promoting the stabilization of KIF3C-mRNA by IGF2 binding protein 1 (IGF2BP1). The prediction that miR-320d inhibits KIF3C expression by targeting METTL3 using the miRmap website, was later confirmed experimentally. Further, a recovery experiment was used to confirm that miR-320d inhibited the progression of prostate cancer. KIF3C was overexpressed in prostate cancer, promoting its growth migration and invasion was induced by miR-320d/METTL3 in an m6A dependent process.

## INTRODUCTION

Prostate cancer (PCa) is currently one of the most common malignancy found across the world, and the second main cause of cancer-related deaths in males [1]. Although, the popularity of prostate-specific antigen (PSA) screening technology has increased the rate of diagnosis and the treatment of prostate cancer, where early prostate cancer can be completely cured, yet about 10% to 20% of the newly diagnosed prostate cancer patients are at their advanced stages [2]. These patients are usually given androgen deprivation therapy (ADT). However, hormone therapy can only last for a median of two years without progression [3]. Therefore, understanding the mechanism of prostate cancer progress is of great significance in treating the disease.

Kinesin superfamily proteins (KIFs) were discovered in 1985 [4]. They are found in eukaryotic cells, and more than 650 members have been discovered till date. KIFs are a class of proteins that move unidirectionally along the microtubules and participate in a variety of intracellular physiological activities, including the transport of organelles and protein molecules, in cell morphology and cytoskeleton dynamics [5, 6]. KIF3 is one of the subfamilies of the KIFs, which includes three members: KIF3A, KIF3B, and KIF3C, and they all are reported to be associated with malignancy. KIF3C is highly expressed in the nervous system and is involved in anterograde axonal transport in mouse neuronal cells [7]. A recent study revealed that KIF3C is an injury specific kinesin that contributes to axon growth and regeneration by regulating and organizing the microtubule cytoskeleton in the growth cone [8–12]. In the tumor diseases, accumulated researches indicated that KIF3C exerted as an oncogene involving in glioma, breast cancer progress by activating PI3K/AKT/mTOR and TGF-β signaling pathway [13, 14]. However, these studies have focused on studying KIF3C in normal mouse cells and other tumor disease, but its research on human prostate cancer needs in-depth studies.

N6-methyl adenosine (m6A) is the most common modification method in mRNA [15, 16]. It adds a methyl group to the mRNA, and then demethylase, catalyzed by N6-methyltransferase removes the methyl group, dynamically regulating the mRNA modification, which affects biological processes such as RNA stability, nucleation, splicing or translation [15]. Studies have shown that m6A RNA modification plays an important role in the pathogenesis of human diseases, and any abnormality in this modification may cause tumorigenesis [17, 18]. Methyltransferase like 3 (METTL3) is a key catalyst in m6A modification and plays a carcinogenic role in a variety of cancers, such as stomach cancer [19], bladder cancer [20], and pancreatic cancer [21]. However, the relationship between METTL3 and prostate cancer is unclear. Additionally, recent evidence suggested that genetic variation in miRNA and its targets may be related to the efficacy of androgen deprivation therapy (ADT) in patients with prostate cancer [22]. Hence, we aimed to explore the relationship between METTL3, KIF3C, and miR-320d.

In our study, we found that the KIF3C level is increased in prostate cancer tissues, and down-regulation of KIF3C can inhibit the proliferation and invasion of prostate cancer cells. Bioinformatics predicted a regulatory relationship between KIF3C and METTL3-mediated m6A modification. Hence, our study aimed to explore the role of the regulatory mechanism of KIF3C, METTL3, and miR-320d in developing prostate cancer.

## RESULTS

### KIF3C is overexpressed in PCa and is negatively correlated with the prognosis

**Figure 1:**
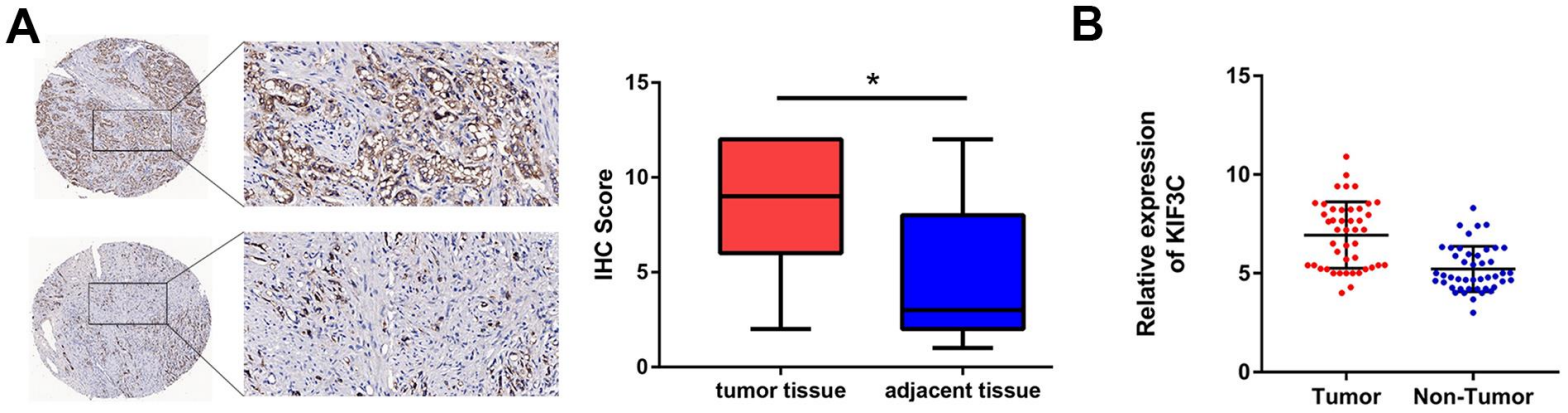
**KIF3C is overexpressed in PCa.** (**A**) Immunohistochemistry of tumor and adjacent tissues was conducted to show KIF3C levels (80 cases). (**B**) RT-qPCR showing KIF3C expression in tumor and adjacent tissues. Data are reported as means ± standard deviation of three independent experiments. *p < 0.05; **p < 0.01.

Firstly, the expression of KIF3C in PCa was explored to figure out its participation in the disease. The immunohistochemistry results showed that the expression of KIF3C was higher in prostate cancer tissues than in the adjacent tissues (Figure 1A). While KIF3C expression was detected in tumor and adjacent normal tissues as well (Figure 1B). The clinical relevance of KIF3C was built on an excel sheet (Table 1), the results indicated that KIF3C is positively with PCa lymph node metastasis and Seminal vesicle invasion.

**Table 1:** The correlation of KIF3C expression with the prostate cancer clinical features.

**Variable**	**Group**	**KIF3C protein expression**			**P value**
		**n**	**High**	**Low**	
Age	<70	45	28	17	0.474
	≥70	35	19	16	
Lymph node metastasis	Positive	17	14	3	0.026
	Negative	63	33	30	
Surgical margin status	Positive	14	10	4	0.289
	Negative	66	37	29	
Seminal vesicle invasion	Positive	26	20	6	0.022
	Negative	54	27	27	
PCa stage	T1	53	29	26	0.105
	T2/T3	27	18	7	
Preoperative PSA	<4	2	1	1	0.038
	4-10	33	14	19	
	>10	45	32	13	
Gleason score	<7	31	15	16	0.035
	7	27	14	13	
	>7	22	18	4	
Angiolymphatic invasion	Positive	20	15	5	0.6576
	Negative	60	32	28	
Biochemical recurrence	Absence	59	34	25	0.7324
	Presence	21	13	8	

### KIF3C silencing prevented proliferation, migration and invasion of PCa cells

**Figure 2:**
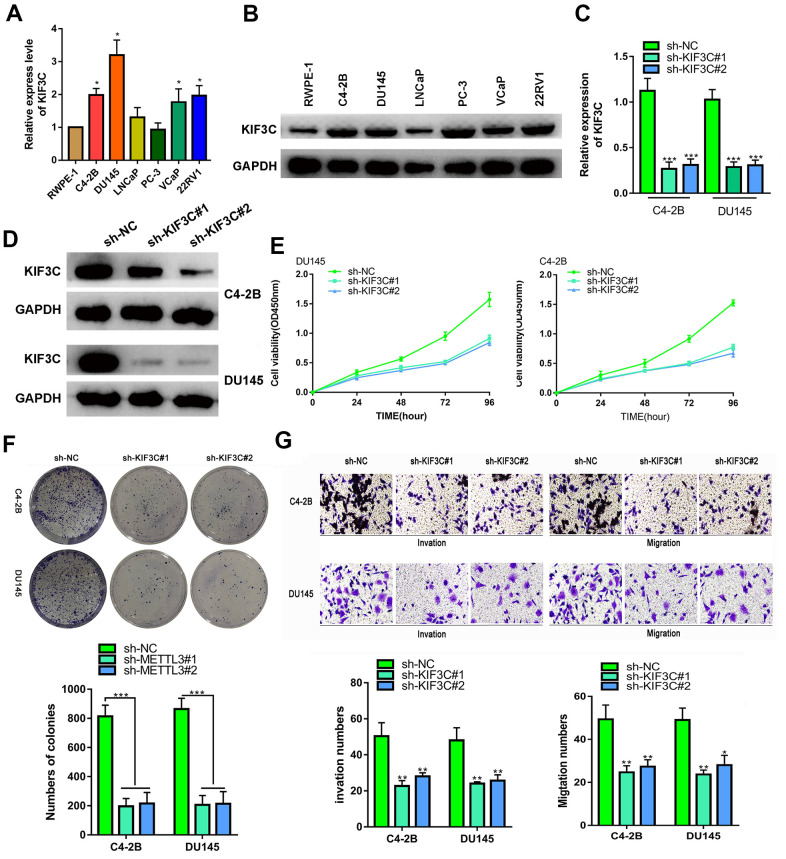
**KIF3C silence prevented the proliferation migration and invasion of PC cells.** (**A**, **B**) RT-qPCR and western blotting showing KIF3C expression in PCa cell lines. (**C**, **D**) RT qPCR and western blotting showing KIF3C knockdown expression in C4–2B and DU145 cells. (**E**, **F**) CCK8 and cloning formation assays showing proliferation after KIF3C knockdown in PCa cells. (**G**) Transwell showing invasiveness and migration capability of PCa cells after knocking down KIF3C. Data are reported as means ± standard deviation of three independent experiments. *p < 0.05; **p < 0.01.

Secondly, the biological function of KIF3C was explored. The KIF3C expression in prostate cancer cell lines was detected by q-RT-PCR and western blotting, the results indicated that KIF3C was overexpressed in the prostate cell lines. (Figure 2A, 2B). KIF3C was silenced in C4–2B and DU145 cells, which expressed the highest KIF3C level (Figure 2C, 2D). Silencing KIF3C reduced growth, migration and invasion of both the PCa cell lines (Figure 2E–2G). Overall, knocking down KIF3C prevented the PCa proliferation, migration and invasion *in vitro*.

### METTL3 facilitated IGF2BP1-regulated stabilization of KIF3C mRNA

**Figure 3:**
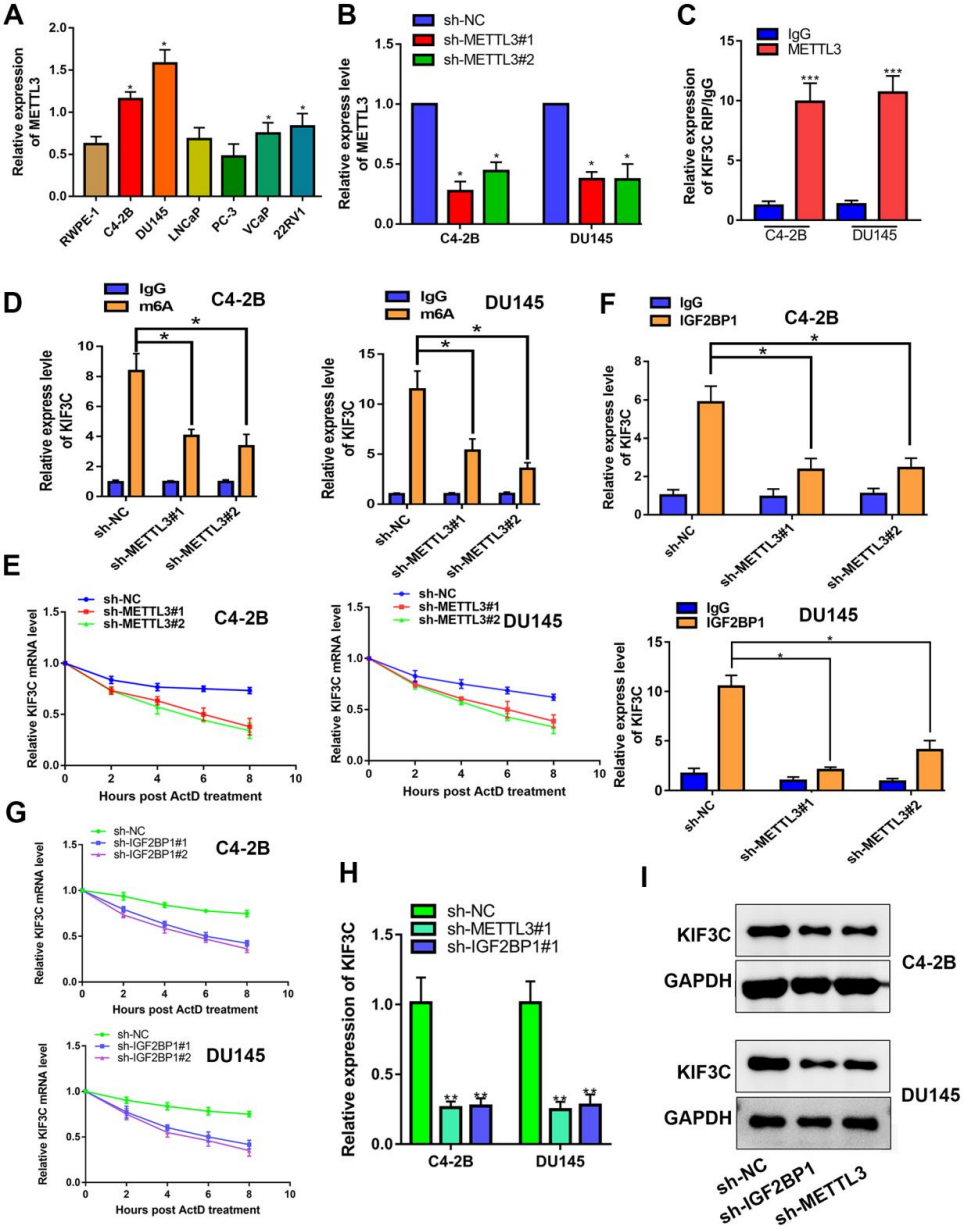
**METTL3 facilitated IGF2BP1-regulated stabilization of KIF3C mRNA.** (**A**) RT-qPCR showing METTL3 expression in PCa cell lines. (**B**) RT-qPCR showing METTL3 knockdown expression in C4–2B and DU145 cells. (**C**) RIP assay was conducted to show the interaction between METTL3 and KIF3C in PCa cells. (**D**) m6A RIP assay was conducted to show the m6A modification on KIF3C mRNA in C4–2B and DU145 cells. (**E**) RT qPCR was conducted to show the stability of KIF3C mRNA after adding ActD. (**F**) RIP assay was conducted to show the effects of the down-regulation of METTL3 on the interaction between IGF2BP1 and KIF3C in C4–2B and DU145 cells. (**G**) RT-qPCR showing the stability of KIF3C mRNA after adding ActD in C4–2B and DU145 cells. (**H**, **I**) RT-qPCR and western blot were conducted to confirm the KIF3C expression under METTL3 or IGF2BP1 silencing. Data are reported as means ± standard deviation of three independent experiments. *p < 0.05; **p < 0.01.

Thirdly, the mechanism of KIF3C upregulation in PCa was interrogated. Using Starbase 3.0 (http://starbase.sysu.edu.cn/), we found that METTL3 can interact with KIF3C. Recent studies have found that METTL3 can catalyze m6A modification of mRNA, thereby upregulating the related gene expression and promoting cancer progression [23]. Therefore, we hypothesized that METTL3 can induce KIF3C expression. The expression of METTL3 in prostate cancer cell lines determined that it is highly expressed in prostate cancer (Figure 3A and Supplementary Figure 1A). The knockout of METTL3 was confirmed in PCa cell lines though RT-PCR (Figure 3B and Supplementary Figure 1B). RIP assay detected a large amount of KIF3C-mRNA in the METTL3 antibody precipitate (Figure 3C). Also, the m6A RIP assay illustrated that silencing METTL3 reduced the m6A modification in KIF3C-mRNA (Figure 3D). The m6A modification could reportedly strengthen mRNA stability. Hence, we examined the effect of METTL3 on the stability of KIF3C-mRNA. RT-qPCR results, after the addition of actinomycin D, showed that METTL3 silencing disrupted the mRNA stability of KIF3C (Figure 3E). Further, through RMBase 2.0 (http://rna.sysu.edu.cn/rmbase/), we found a binding site for m6A in KIF3C and predicted that IGF2BP1 can interact with KIF3C (Supplementary Figure 2). Studies have shown that m6A modification promotes the binding of IGF2BP1 to the target mRNA, thereby promoting the stability of mRNA [24]. Therefore, we tested whether METTL3 can stabilize KIF3C-mRNA by IGF2BP1 or not. RIP assay confirmed that IGF2BP1 interacts with KIF3C-mRNA, and METTL3 silencing reduces KIF3C-mRNA enrichment in IGF2BP1 precipitation (Figure 3F). The half-life of KIF3C-mRNA was reduced after down-regulating IGF2BP1 (Figure 3G). Also, silencing METTL3 or IGF2BP1 reduced KIF3C-mRNA and protein expression in PCa cells (Figure 3H, 3I).

### METTL3 is the target gene of miR-320d

**Figure 4:**
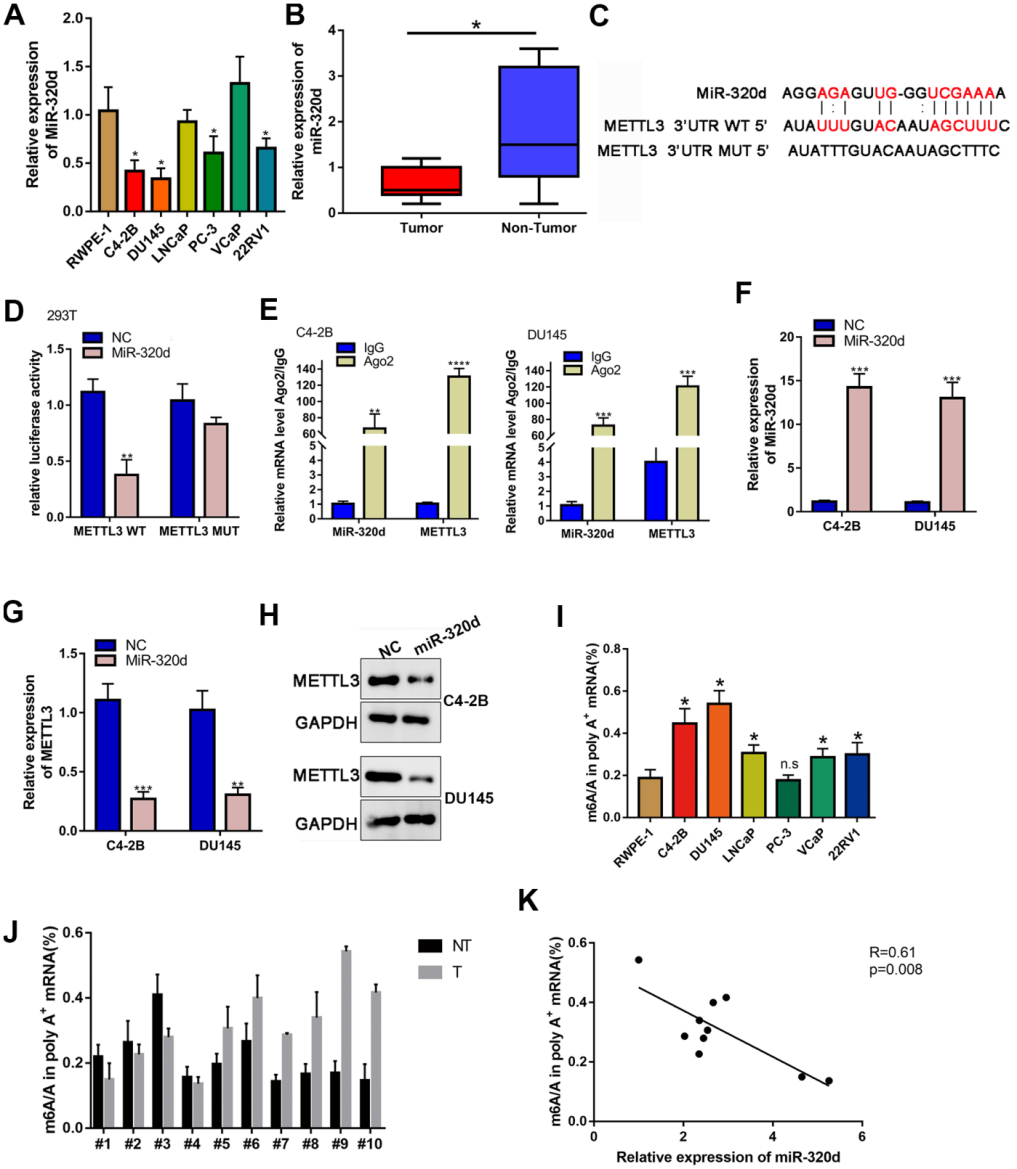
**METTL3 is the target gene of miR-320d.** (**A**) RT-qPCR showing miR-320d expression in PCa cell lines. (**B**) Binding sites between miR-320d and METTL3 from Starbase 3.0 and the mutant sites. (**C**) Luciferase assay of cells transfected with pmirGLO-3’UTR reporter of METTL3 in the miR-320d overexpressing 293T cells. (**D**) RIP assays were conducted to show the interaction between miR-320d and METTL3. (**E**, **F**) RT-qPCR and western-blot showing expression of miR-320d and METTL3 in the miR-320d overexpressing PCa cells. (**G**, **H**) RT-qPCR and western blot were conducted to show KIF3C expression under indicated transfection. Data are reported as means ± standard deviation of three independent experiments. *p < 0.05; **p < 0.01. (**I**, **J**) Quantification of m6A levels in mRNA extracted from pancreatic cancer cell lines and PCa tissues. (**K**) The correlation of the miR-320d expression and m6A level in the PCa tissues. *p < 0.05; **p < 0.01.

The upstream mechanism of METTL3/KIF3C in PCa is discussed further. It is believed that miRNAs are regulators of gene expression, and they participate in cancer progression [25]. The miRmap (https://mirmap.ezlab.org/) was explored to find potential miRNAs that target METTL3. The miR-320d interacted effectively with METTL3 and was chosen for further research. The low expression of miR-320d in PCa cell lines and tissues were confirmed by qPCR (Figure 4A, 4B). The binding sequences on METTL3 for miR-320d and the mutated sites are shown in Figure 4C. Overexpression of miR-320d significantly reduced the luciferase activity of METTL3 WT but did not reduce the luciferase activity of METTL3 Mut (Figure 4D). RIP assay identified co-immunoprecipitation of miR-320d and METTL3 in two PCa cell lines by the Ago2 antibody (Figure 4E). Next, miR-320d was also overexpressed in two PCa cell lines using miR-320d mimic (Figure 4F). Overexpression of miR-320d down-regulated the mRNA and protein expression of METTL3 (Figure 4G, 4H). Furthermore, the m6A level of PCa cells and tissue were higher than normal prostate tissue and cells (Figure 4I, 4J). Also, the expression of miR-320d was negatively correlated with m6A level in PCa tissues. (Figure 4K).

### miR-320d inhibited PC progression through METTL3/KIF3C

**Figure 5:**
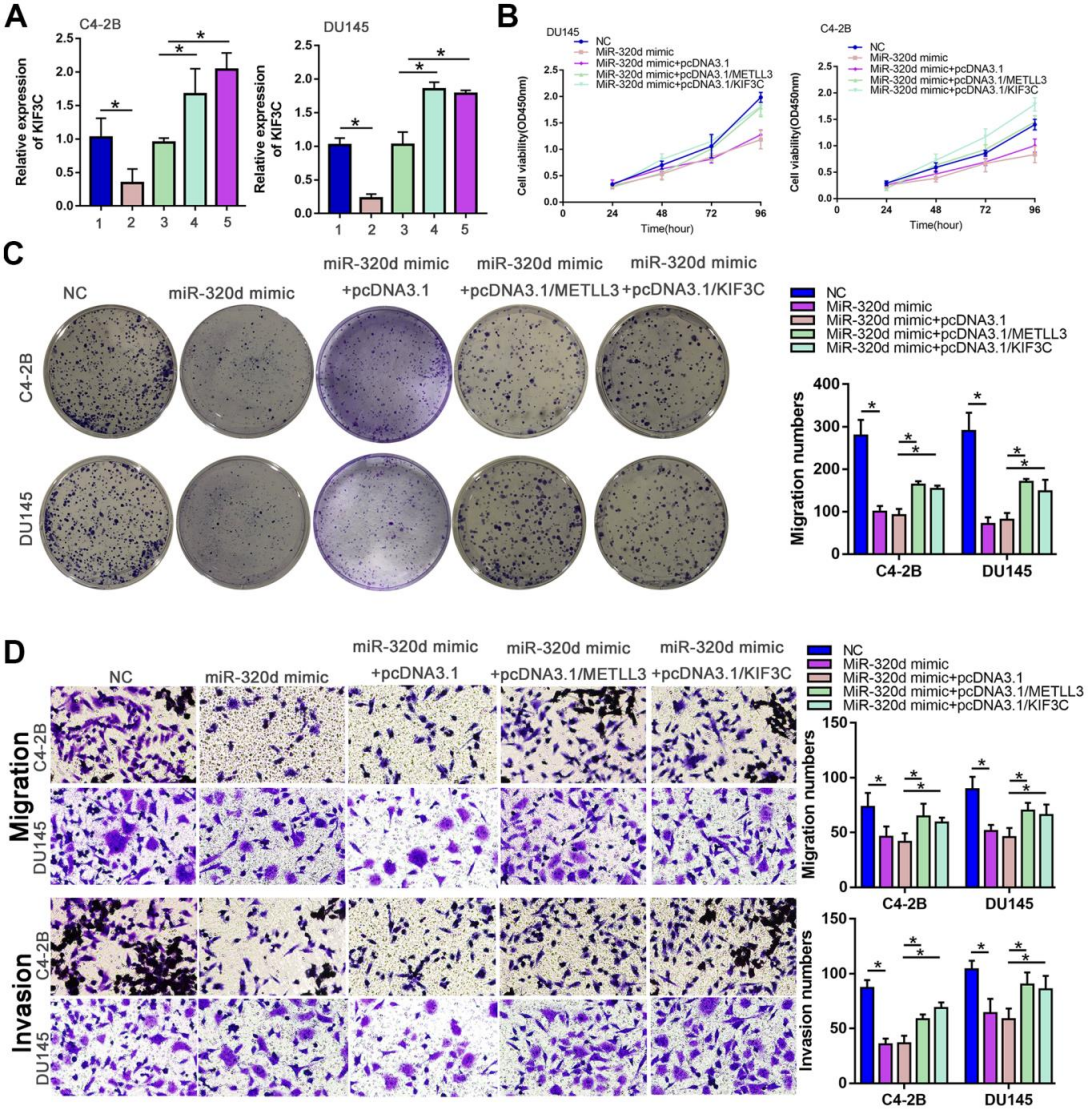
**MiR-320d inhibited PC progression through METTL3/KIF3C.** C4–2B and DU145 cells were transfected with NC mimic, miR-320d mimic, miR-320d mimic+pcDNA3.1, miR-320d mimic+pcDNA3.1/METTL3, or miR-320d mimic + pcDNA3.1/KIF3C, respectively. (**A**) RT-qPCR showing KIF3C expression in indicated group PCa cells. (**B**, **C**) CCK-8 and colony formation were conducted to show the proliferation of PCa cells. (**D**) Transwell showing invasion and migration ability of PCa cells. Data are reported as means ± standard deviation of three independent experiments. *p < 0.05; **p < 0.01.

Finally, we confirmed if miR-320d can regulate PCa progress by recovery experiment. The expression of KIF3C was lowered in the case of overexpression of miR-320d, and restored by overexpression of METTL3 or KIF3C (Figure 5A). The proliferation, invasion, and migration of PCa were retarded by miR-320d mimic and rescued by the ectopic expression of METTL3 or KIF3C (Figure 5B–5D).

### KIF3C silence prevented the proliferation of PC cells *in vivo*

**Figure 6:**
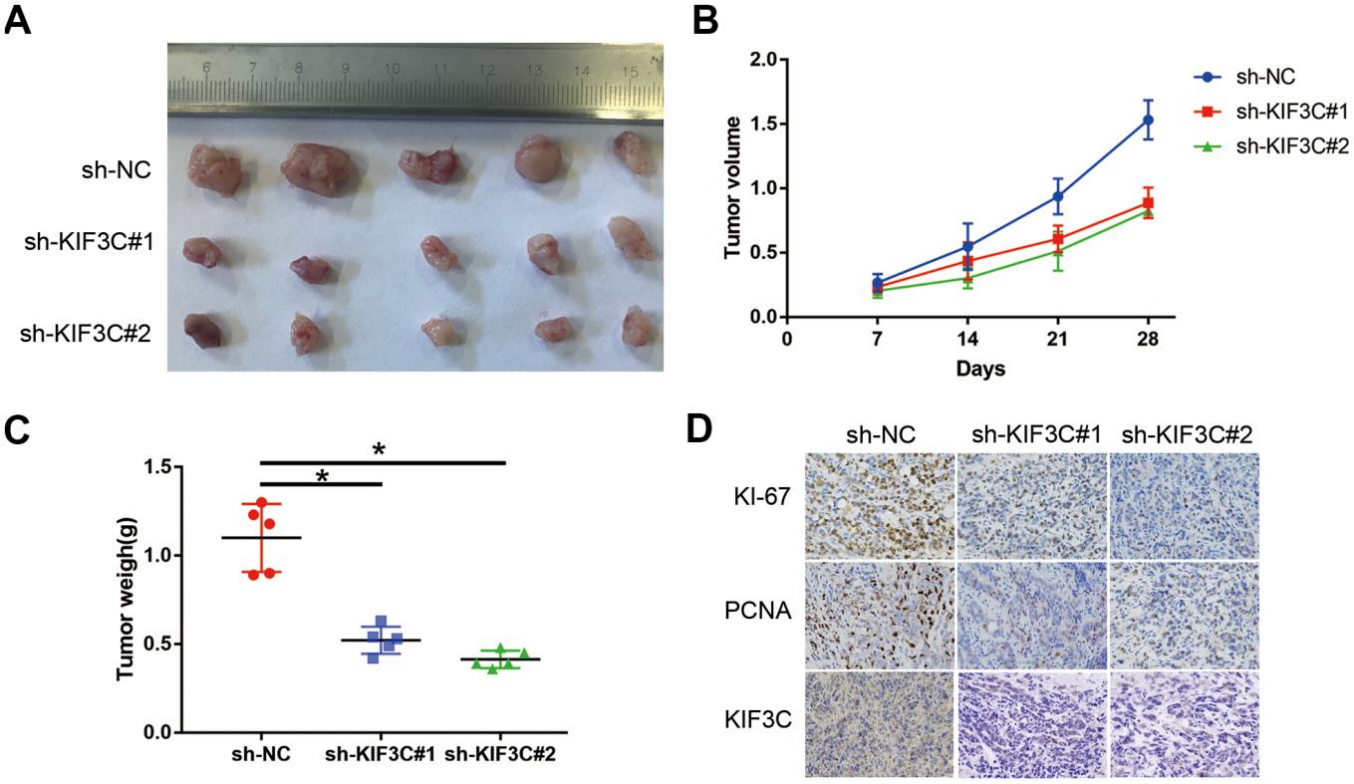
**KIF3C silence prevented the proliferation of PC cells *in vivo*.** (**A**) Represent image of nude mouse tumors (n=5). (**B**) The implanted tumor volume of the KIF3C knockdown PCa cells nude mouse. (**C**) The implanted tumor weight of the KIF3C knockdown PCa cells nude mouse. (**D**) Typical IHC staining images showing Ki-67, PCNA and KIF3C of the implanted tumors. Data are reported as means ± standard deviation of three independent experiments. *p < 0.05; **p < 0.01.

To further evaluate the effects of KIF3C on tumorigenesis *in vivo*, we conducted a xenograft tumor model by subcutaneously injecting the C4-2B PCa cells. The representative xenograft tumors from each group at 5 weeks were photographed (Figure 6A). The weigh and volume of the implanted tumors showed that KIF3C knockdown PCa cells exerted more slower growth ability (Figure 6B, 6C). Immunohistochemistry (IHC) staining analysis of the correlation of KIF3C expression of the KI-67, PCNA and the two markers were significantly downregulated in the KIF3C silenced groups (Figure 6D).

## DISCUSSION

The occurrence and development of prostate cancer are closely related to the abnormal expression of oncogenes [26]. Earlier studies have found that the kinesin family’s proliferation, invasion, and migration are closely related to tumors [27]. KIF3C, a newly discovered prostate cancer molecule in our study, also exists in other cancers [14, 28]. KIF3C is reported to express highly in breast cancer and promote tumor progression [14]. Similarly, we found that KIF3C is highly expressed in prostate cancer and is closely related to the prognosis of the disease as well. Functional tests have also shown that it can promote proliferation, invasion, and migration, but the reason behind its high expression and the molecular mechanism of promoting tumorigenesis and development remains unclear.

In eukaryotes, the 5′-cap and 3′-polyA modifications play a very important role in regulating transcription, and the internal modifications of mRNA help in maintaining its stability [29]. The most common internal modifications of mRNA include N6- methyladenosine (m6A), N1- methyladenosine (m1A), 5- methylcytosine (m5C), and so on. The m6A modification can accelerate the processing time of the mRNA precursors, speed of mRNA transport, and nucleation in cells [30, 31]. Therefore, we used bioinformation based predictions to find the presence of m6A modification in KIF3C and hypothesized that KIF3C overexpression in prostate cancer is mediated by its m6A modification. Later, RIP assays explored that m6A can bind to KIF3C and regulate its expression. The m6A methylase METLL3 has been studied in a variety of tumors and is closely related to the tumor’s high invasive capacity and rapid appreciation [32, 33]. The expression of m6A and KIF3C decreased after knocking down METLL3. This conclusion is consistent with the previous assumptions. Further, through RIP assay and bioinformatics prediction, we found that IGF2BP1 can be used as a methylation recognition enzyme for m6A modification of KIF3C. The IGF2BP1 family is reported in the literature, as an m6A methylase and promoter of cancer [24]. Thus, we tested the expression and stability of KIF3C after knocking down IGF2BP2. The results showed that the expression and stability of KIF3C are regulated by IGFBP1, and this process is m6A-dependent.

The role of miRNA in cancer is widespread [34]. It is unclear whether miRNAs can participate in the regulation of m6A or not. We found that METTL3 can be regulated by miRNA-320d through bioinformatics prediction and further studied the regulatory relationship between miR-320d and METTL3. Expression and dual fluorescence experiments showed that miR-320D can target METTL3 and regulate its expression. Further confirmation by molecular and cellular function tests showed that miRNAs can inhibit PCa progression through METTL3 and KIF3C.

In conclusion, KIF3C was overexpressed in the prostate cancer and exerted as an oncogene involving in promoting proliferation and metastasis, which was regulated by METTL3 in m6A modification dependence.

## MATERIALS AND METHODS

### Tissue specimen

The Specimens and the clinical-pathological data collection were approved by the Institutional Research Ethics Committee of the affiliated hospital of Guizhou Medical University. The IHC analysis of prostate cancer was provided by the affiliated hospital of Guizhou Medical University. The correlation between KIF3C and prostate cancer patients’ clinical features was analyzed and confirmed before specimen collection. A total of 80 patients’ prostate cancer tissues and adjacent tissues were collected between 2015 to 2019 from the urology department of the affiliated hospital of Guizhou Medical University for immunohistochemical detection. All the patients’ diagnostic criteria were classified as prostate cancer, according to the World Health Organization’s diagnostic criteria. The Specimen and clinical pathological data collection were approved by the Institutional Research Ethics Committee of the affiliated hospital of Guizhou Medical University (approval no. GMU2019-058). All patients provided written informed consent prior to enrollment in the study.

### Cell culture

The human PCa cell lines, C4-2B (prostate cancer), DU145 (prostate cancer brain metastases), LNCaP (prostate cancer Lymph node metastasis), PC-3 (prostate cancer bone metastases), RWPE1 (prostate cancer), 22RV1 (prostate cancer), VCaP (vertebral metastasis) were acquired from the Shanghai Chinese Academy of Sciences cell bank (China). All the cells were cultured in high glucose DMEM medium (Hyclone) with 10% fetal bovine serum (Gibco) and grown in a cell culture incubator with 5% CO_2_ at 37° C.

### Cell transfection

The cells were plated on to a six-well plate and grown until the confluency reached 50%. For transfection, siRNA, and Lipofectamine 2000 were diluted in serum-free medium, mixed and kept separately for a few minutes. Next, the transfection reagent and the siRNA diluent were mixed by pipetting 3–5 times gently and allowed to stand for a few minutes at room temperature. This mix was then added to the six-well plate, shaken, and incubated at 37° C with 5% CO_2_ for 24–48 h. Fresh medium was added after 4–6 h of transfection. According to the instruction, the concentration of the miRNA or siRNA is 0.05nmol/ml and the transfection 48h for RNA and protein extracting. The sequences of short hairpin RNA (shRNA) interference are as follows: sh-KIF3C#1: 5′-CGAACCGAGCCAAGAACAUUC UCUUGAAAUGUUCUUGGC UCGGUUCGGGG-3′, sh-KIF3C#2: 5′-GCUGCCCAAGACCUUCACUUCUCUUG AAAGUGAAGGUCUUGGGCAGCGGG-3′, a scramble sequence was used for negative control (NC). SiRNA of METTL3: CTGCAAGTATGTTCACTATGA, siRNA of IGF2BP1: ACGCTTAGAGATTGAACATTC. And these shRNA or siRNA were purchased from Ribobio (Guangzhou, China).

### Quantitative real-time polymerase chain reaction (RT-qPCR)

RNA from each group was extracted using Trizol Reagent (Invitrogen, CA, USA) according to the instructions. Then, cDNA was synthesized using the PrimeScript RT reagent kit (Takara) according to the manufacturer’s protocol. Real-time quantitative PCR was performed using the Powerup SYBR Green PCR Master Mix (Life Technologies).

### Cell viability analysis

Cell Counting Kit-8 (Beyotime, China) was used to detect cell proliferation capacity. As per the instructions, 100 uL of 2x10^3^ cells were seeded into a 96-well plate, and 10 μL of CCK8 solution was added to each well. Absorbance values were measured at different time points of 0, 24, 48, 72, and 96 h.

### Colony formation assay

Each group of cells, in their exponential growth phase, were seeded into a six-well plate at 1000 cells per well, and then 2 mL of 10% FBS supplemented DMEM medium was added to each well, mixed and incubated for 7 to 14 days. Later, it was fixed with 4% paraformaldehyde and stained with 0.5% crystal violet.

### Transwell assay

Corning’s 24-well Transwell system was used for the invasion and transfer experiments. 1x10^5^ cells were mixed with 200 μL of serum-free medium and added to each of the upper chambers. Subsequently, 700 μL of 10% FBS medium was added to the lower chamber. After 24–48 h of incubation, the upper chamber was removed and stained with 0.2% crystal violet. The migrated cells in the lower chamber were photographed and counted.

### Western blot analysis

Cells from each group were lysed using RIPA (Beyotime, China) to extract the protein. After quantifying the protein using BCA reagents (Beyotime, China), the sample was re-diluted to the same concentration, boiled, and stored in a refrigerator at –20° C until further use. SDS-PAGE gel with 10% separation gel and 5% concentrated gel was prepared using Solarbio’s (China) reagents, according to the manufacturer’s procedure. Samples were electrophoresed, transferred, and then finally exposed. The protein content we add each time is 30μg.

### Reporter gene transfection and luciferase activity assay

The cells were seeded in a six-well plate, and after reaching 80% –90% confluency, they were co-transfected with a luciferase reporter gene, miR-320d, or METTL3 containing TA promoter, for 4–6 h using LipofectAMINE reagent (Invitrogen). After 24 h of transfection, the luciferase activity in the cell extract was measured using a double luciferase gene detection kit (Beyotime, China).

### RNA immunoprecipitation (RIP) assay

The Magna RIP™ Quad RNA-Binding Protein Immunoprecipitation Kit (Millipore, MA, USA) was used to analyze Protein-RNA Interaction. The total RNA was mixed with antibodies (against IgG, m6A, IGF2BP1, or Ago2) or rabbit IgG-binding protein A/G magnetic beads in 1x IP buffer. The RNA of interest was immunoprecipitated with the beads. All the antibodies were provided by Magna.

### IHC analysis

The expression of KIF3C was detected by immunohistochemical staining. The tissue was cut into 3um slice and then dewaxed. Next, these sections were incubated with rabbit anti-KIF3C monoclonal (1: 200; 14333–1-AP; Proteintech, IL, USA) antibody at 4° C overnight. After washing three times with PBS, each section was incubated with goat anti-rabbit IgG for 30 min and then developed using 3,3’ diaminobenzidine (DAB).

### mRNA stability assay

We used 5 mg/mL Actinomycin D (Act D; Sigma Aldrich (MO, USA) to test the mRNA stability in C4–2B and DU145 cell lines as per the manufacturer’s protocol. At different time points, cells were harvested. Subsequently, RNA from each cell sample was extracted and RT-qPCR was performed.

### *In vivo* assay

For the proliferation assays, C4-2B cells of KIF3C knockdown, negative control (1×10^6^/200μl) were subcutaneously injected into BALB/c nude mice. The tumors were dissected and weighed (4–6 weeks old, male). The animal experiments were approved by the Animal Care Committee of The First Affiliated Hospital of Guizhou medical University. The animal experiments were approved by the Animal Care Committee of The First Affiliated Hospital of Guizhou medical University (approval no. 2006369).

### Statistical analysis

The experimental data was repeated thrice and expressed as mean + SD, and all the data were processed by Prism7.0 analysis. A comparison between the groups was performed using Student’s t-test, ANOVA and Person analysis. P-value of less than 0.05 indicated a statistically significant difference.

### Data availability

Data supporting the findings of this study are available from the corresponding author upon request.

## Supplementary Material

Supplementary Figures
