# Supplementary material for: METTL3-mediated m6A modification of KIF3C-mRNA promotes prostate cancer progression and is negatively regulated by miR-320d
Source: Aging (Albany NY). 2021 Sep 19;13(18):22332–44. doi: 10.18632/aging.203541 (PMC8507285; doi:10.18632/aging.203541)
Supplement: Supplementary Figures [file aging-13-203541-s001.pdf]

SUPPLEMENTARY FIGURES

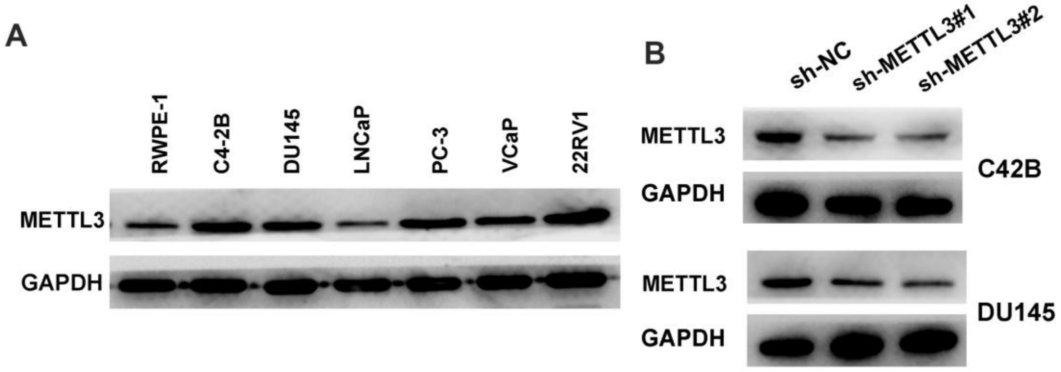

Supplementary Figure 1. (**A**) Western blotting showing METTL3 expression in PCa cell lines. (**B**) Western blotting showing METTL3 knockdown expression in C4–2B and DU145 cells.

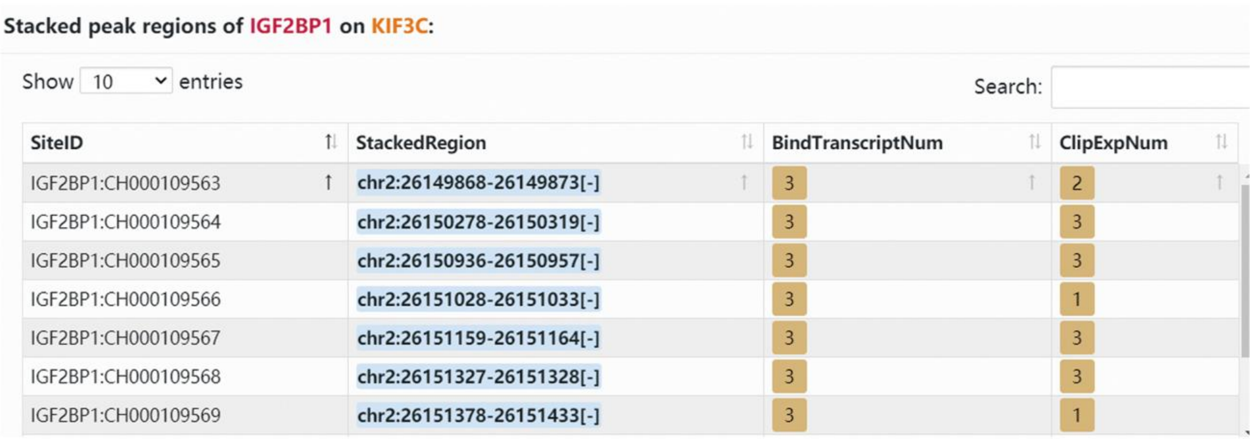

Stacked peak regions of IGF2BP1 on KIF3C:

Show 10 entries

Search:

SiteID	StackedRegion	BindTranscriptNum	ClipExpNum
IGF2BP1:CH000109563	chr2:26149868-26149873[-]	3	2
IGF2BP1:CH000109564	chr2:26150278-26150319[-]	3	3
IGF2BP1:CH000109565	chr2:26150936-26150957[-]	3	3
IGF2BP1:CH000109566	chr2:26151028-26151033[-]	3	1
IGF2BP1:CH000109567	chr2:26151159-26151164[-]	3	3
IGF2BP1:CH000109568	chr2:26151327-26151328[-]	3	3
IGF2BP1:CH000109569	chr2:26151378-26151433[-]	3	1

Supplementary Figure 2. Bioinformatic analysis the KIF3C RNA binding protein IGF2BP1.
